# Male Breast Cancer: A Rare Entity

**DOI:** 10.31729/jnma.3662

**Published:** 2018-08-31

**Authors:** Swotantra Gautam, Brikha Raj Joshi, Shailesh Adhikary, Sudeep Regmi, Anju Pradhan

**Affiliations:** 1 B P Koirala Institute of Health Sciences, Dharan, Nepal; 2 Department of Surgery, B P Koirala Institute of Health Sciences, Dharan, Nepal; 3 Department of Pathology, Lumbini Medical College and Teaching Hospital, Tansen, Nepal; 4 Department of Pathology, B P Koirala Institute of Health Sciences, Dharan, Nepal

**Keywords:** *female breast carcinoma*, *infiltrating carcinoma*, *male breast carcinoma*

## Abstract

Male breast carcinoma is a rare malignancy (<1% of all breast carcinomas, 0.2% of all male malignancies). Its common histopathological type is infiltrating carcinoma, not otherwise specified. Three male patients aged 56 (stage -IIIB), 64 (T4bN0M0) and 78 (T2N0MO) years presented with a breast lump within a year. Their hematological and biochemical parameters were within normal limits. Two of them had palpable regional lymph nodes. Male breast carcinoma occurs in older males as in our cases. Two cases showed infiltrating ductal carcinoma, not otherwise specified on histopathological evaluation, and one showed special type with apocrine differentiation. Their two-year follow-up was uneventful after modified radical mastectomy and chemotherapy. Male breast carcinoma is associated with risk factors different from and overlapping with female breast carcinoma. Male breast carcinoma differs from female breast carcinoma on clinical presentation, biological behaviour and prognosis. Male breast carcinoma as a separate clinical entity with its own biological behaviour is manageable by surgery and chemotherapy.

## INTRODUCTION

Male Breast Carcinoma (MBC) is a relatively rare malignant disease, and accounts for about less than 1% of all breast carcinomas, and 0.2% of all malignancies in males.^1,2^ Approximately 1.2 cases are affected in 100,000 males,^3^ and are associated with mutation of BRCA1 and BRCA2 genes, with BRCA2 mutation being the most common.^4^

Most common histopathological type of male breast carcinoma is Infiltrating Carcinoma, Not Otherwise Specified (NOS). However, all other special types of breast carcinomas as in females can occur in male breast carcinomas.^5^

Compared to the female breast carcinoma, male breast carcinoma presents with a more advanced disease with around half of the patients presenting with axillary lymph node metastasis at first visit, and tumor size of >2.0cm.^6^ We present three cases of MBC with their demography and management.

## CASE REPORT

### CASE 1

A 56-year-old man presented with a history of lump in the right breast for 8 months, insidious in onset, rapidly progressive associated with a swelling in the right axillary and supraclavicular region. Past history, family history and personal history were not significant. General physical examination was normal. On local examination, a hard, oval lump 5x4 cm was palpated in the right breast in the centre involving all the four quadrants, along with enlarged ipsilateral axillary lymph nodes each measuring 1x1 cm. Clinically it was stage IIIB. FNAC reported malignancy of breast. He was given two cycles of pre-operative chemotherapy with (Inj.

**Figure 1.**
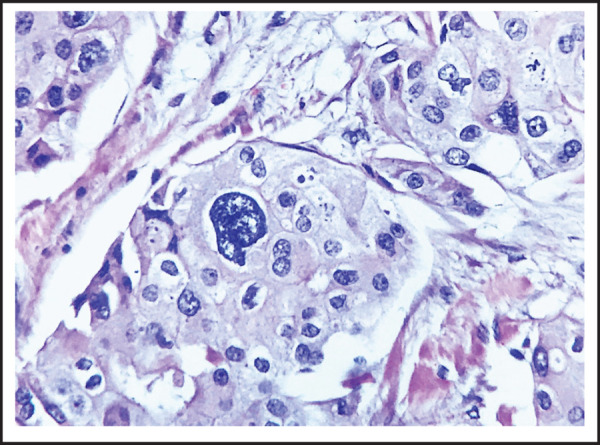
Infiltrating ductal carcinoma with apocrine differentiation. Tumor cells are showing moderate to marked pleomorphism. There is presence of round to vesicular nuclei, irregular nuclear membrane, visible to prominent nucleoli and moderate amount of granular eosinophilic cytoplasm.

Cyclophosphamide 1000mg, Inj. Adriamycin 100mg, 5FU 1000mg and Inj. Filgastron 30U). The patient was then planned for a right-sided Auchincloss modified mastectomy. Intraoperative findings were right breast lump around the nipple areola complex involving all quadrants, pectoralis major muscle, and presence of matted fixed axillary lymph nodes. Histo-pathological examination showed Invasive breast carcinoma, NOS with Apocrine differentiation (Figure 1).

It was with associated with Nottingham Prognostic Index (NPI Score) 5 (Moderate prognosis), pTNMNx (AJCC 8^th^ Edition). He is on regular follow up and he also received 3 cycles of postoperative chemotherapy.

The patient was doing well till 2 years after operation.

### CASE 2

A 64-year-old man presented with a history of lump in the right breast for 2 years, and it was increasing in size since its appearance. Past history, family history and personal history were not significant. His vitals were stable. On physical examination, a firm lump measuring about 8x5cm was palpated in the outer lower quadrant of the right breast. It was fixed to the chest wall and skin. The clinical stage was T4bN0M0. Fine needle cytology showed malignancy of breast. He was planned for a right-sided modified radical mastectomy. Before the surgery patient received two cycles of chemotherapy (Cyclophosphamide, Adriamycin and Filgastron). At operation lump of size 7x6 cm was removed from right upper outer quadrant. Level I and level II axillary lymph nodes were slightly enlarged. Histo-pathological examination showed invasive breast carcinoma, NOS, Grade 2 (T2N1M0) (AJCC 7^th^ edition) with NPI score of 8. He received 3 cycles of postoperative chemotherapy (Inj. Cyclophosphamide 1000mg, Inj. Adriamycin 100mg, 5FU 1000mg and Inj. Filgastron 30U). He was on regular follow up, 6 monthly for next 2 years post-surgery, and he is doing well.

### CASE 3

**Figure 2.**
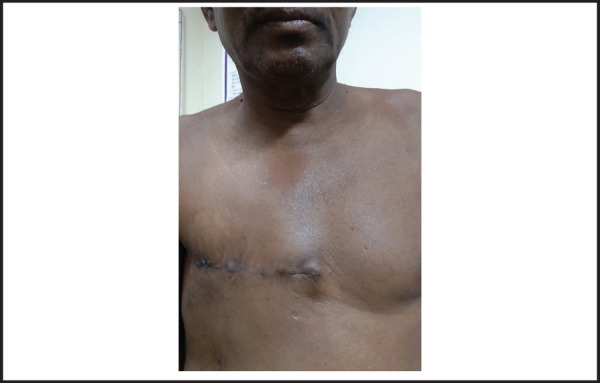
Post-operative picture of the right breast carcinoma on follow up visit.

A 78-year-old man presented with a history of painless swelling in the left breast for 1 month. On general examination there was bi-pedal edema and patient gave history of shortness of breath. Chest x-ray showed cardiomegaly. Echocardiography was done and it was suggestive of right atrium, right ventricle dilatation, hypertrophied right ventricle, mild tricuspid regurgitation, pulmonary arterial hypertension with grade I diastolic dysfunction. On physical examination, a non-tender lump measuring about 3x2 cm was palpated just beneath the left nipple; it was mobile and not adhered to underlying structures. The clinical stage was T2N0M0. Fine needle cytology reported carcinoma of breast. He underwent a right-sided Auchincloss modified mastectomy. Operative findings were lump of size 3*2cm with enlarged axillary lymph nodes at level II and I. Histo-pathological examination showed invasive breast carcinoma, NOS, Grade 2 with NPI score of 8. He received two cycles of postoperative chemotherapy. He was followed for 2 years post surgery, which was uneventful. Post-operative photograph (Figure 2).

## DISCUSSION

Male breast carcinoma is a malignancy occurring in older males, and has a rare incidence.^6^ With the advancement of early diagnosis and treatment protocols, incidence of female breast cancer is declining. In contrast, cases of MBC are increasing steadily and the improvement of clinical outcome is not promising as in female breast carcinoma (FBC).^7^

Studies have revealed relation of male breast carcinoma with Klinefelter's syndrome, gynecomastia, first-degree relatives with male breast carcinoma, hyperestrogenism, diabetes, alcohol intake, physical inactivity, increased BMI, orchitis/epididymitis, Lynch Syndrome, and Li-Fraumeni Syndrome.^2,7,8^ Several other risk associations are to be unfolded as in female breast carcinoma.

All three cases of our study showed infiltrating ductal carcinoma, NOS on histopathological evaluation, and one showed anapocrine differentiation.

Salient features of all three cases are given (Table 1).

**Table 1:** Salient features of all 3 cases of MBC.

Case	Age	Laterality	Size	LN metastasis	Histopathology	Ancillary Investigations
1	56yrs	Right	5×4cm	Present (Ipsilateral Axillary and supraclavicular)	Infiltrating Carcinoma, NOS with Apocrine differentiation	Not Done
2	64yrs	Right	8×5cm	None	Infiltrating Carcinoma, NOS	Not Done
3	78yrs	Left	3×2cm	Present (Ipsilateral Axillary)	Infiltrating Carcinoma, NOS	Not Done

However, special types of breast carcinomas can occur in male breast carcinoma as in female breast carcinoma.^8–10^ Lymph node metastasis was seen in two out of three (66%) of our cases. In a case series of 229 patients, lymph node metastasis was seen in 57% of the patients with male breast carcinoma.^2^

In the past, MBCs were considered as similar to female breast carcinomas, and the clinical management is derived from the treatment of female breast carcinomas. Nowadays, studies have pointed out male breast carcinomas as a different biological entity with differences in etiology, biological behaviour and prognosis, compared to female breast carcinomas.^6^

Based on immune histochemical expression profiles, male breast carcinomas can be classified into following molecular subtypes: Luminal A, Luminal B, Triple Negative and Basal Like (Unclassified). ^3,11^ Compared to FBCs, MBCs present with lower histologic grade and increased oestrogen receptor/progesterone receptor positivity.^6^ However, these ancillary investigations were not done in any of our cases. Male breast carcinoma is a rare entity occurring in older men, and is associated with

many risk factors different from, and overlapping with female breast carcinoma. Many differences regarding clinical presentation, biological behaviour and prognosis are seen in comparison to female breast carcinoma. Thus, male breast carcinoma should be taken as a separate entity with different biological behaviour, from that of female breast carcinoma and our cases were managed by surgery and chemotherapy.
